# The Relationship Between Religious Health Fatalism and Healthy Lifestyle Behaviors of Earthquake Victims: The Example of Türkiye

**DOI:** 10.1007/s10943-024-02243-w

**Published:** 2025-01-08

**Authors:** Fatma Tanrıkulu, Rümeysa Demir, Mustafa Demir

**Affiliations:** 1 Department of Nursing, Faculty of Health Sciences, Sakarya University of Applied Sciences, Akyazı, Sakarya, PA 54400 USA; 2 Department of Medical Services and Techniques, Akyazı Vocational School of Health Services, Sakarya University of Applied Sciences, Akyazı, Sakarya, PA 54400 USA

**Keywords:** Earthquake victim, Fatalism, Health, Behavior, Türkiye

## Abstract

Earthquakes are natural disasters that cause physical, psychological, social, and environmental damage. Due to the intense psychological impact, victims of earthquakes may associate a fatalistic approach with religion as a mechanism for seeking protection. The aim of this study was to determine the relationship between religious health fatalism and healthy lifestyle behaviors among earthquake victims. A comparative analysis was conducted based on sociodemographic factors to compare average scores. A total of 175 individuals who were affected by the earthquakes that occurred in Türkiye in 2023 participated in this descriptive, cross-sectional, and correlational study. The total Religious Health Fatalism Questionnaire score of the earthquake victims was 54.89 ± 14.68 and Healthy Lifestyle Behaviors Scale-II score was 130.14 ± 19.68. Significant correlations were found between religious health fatalism and healthy lifestyling behaviors of the participating individuals (p < 0.05). It was determined that the independent variables explained 35.7% of the RHFQ score (R^2^ = 0.357) and 16% of the HLBS-II scale score (R^2^ = 0.160). Earthquake victims were found to have higher than average levels of religious health fatalism.

## Introduction

Throughout history, fatalistic beliefs have come to be seen as related to believing in God. Therefore, the role and responsibility of a person for the fulfillment of his or her own will over his or her life has been a subject of debate (Kılıç & Malak, 2022). In the context of religious belief, many people believe that fatalism plays a crucial role in a person's life. Especially with regard to events that affect human beings, they say that these events are created by God and do not depend on human will. Certain religious functionaries claim that destiny is a function of human will, and that human beings are in control of their own destiny. (Kaya & Bozkur, 2017). Fatalism is a multidimensional psychological concept. It is also characterized by emotions that have negative connotations, such as worthlessness, weariness, and hopelessness. (Kizilarslan & Yıldız, 2023). Those who adopt a fatalistic approach accept that the course of life is preordained and believe that a person will not be able to change their destiny, even if they try to act. (Abbott, 2020).

Unpredictable natural catastrophes have a negative impact on people's physical, social, and mental health by disrupting society's usual way of life. Although earthquakes initially destroy buildings in the geographical location where they occur, the real destruction occurs in the lives and psychological states of individuals. Experiencing an earthquake resulting in widespread destruction is a significant traumatic event in a person’s life. Earthquake victims experience traumatic stress as a result of their losses, injuries, changes to their living environment, and financial difficulties. (Alsamara et al., 2024; Kurt & Gülbahçe, 2019; Ren & Pak, 2022). Due to its strong psychological impact, the victims of an earthquake may associate the fatalistic approach with religion as a mechanism to find protection. (Hussain et al., 2011).

In a study conducted in the field of theology, it was emphasized that as a response to circumstances that put a person in a difficult situation, such as malice, injustice, and natural disasters, fatalistic attitudes were displayed along with a religious approach by the person to make sense of these circumstances and to continue their lives. The results of some studies conducted after the Great Marmara Earthquake in Türkiye in 1999 showed that a large majority of those affected believed that the earthquake was caused by God, that it was a punishment inflicted by God, and that there is nothing to be done about earthquakes. (Yilmaz, 2023; Kula, 2002). Thus, religiously oriented fatalistic perceptions may influence the way in which disaster victims take measures against future disasters, deal with the consequences of disasters in such cases, or guide their behavior in the post-disaster period. (Kılıç & Malak, 2022).

It has been suggested that fatalism plays an important role in determining individual actions in areas ranging from economics to occupational preferences, and from health practices to natural disaster preparedness. Some research has been concerned with the explanation of the relationship between fatalism and behaviors in the context of health. In the relevant literature, the effects of fatalism on the behaviors of individuals to maintain a healthy lifestyle, to diagnose cancer at an early stage, to comply with the treatment of chronic diseases, and to protect themselves against diseases such as cancer, diabetes, and HIV/AIDS have been studied. (Dayapoglu et al., 2021; Hamed & Daniel, 2017; Hess & Mbavu, 2010; Ningsih et al., 2022). Studies were also conducted during the COVID-19 pandemic to determine how many people held fatalistic beliefs and how fatalism affected pandemic health behaviors. (Akesson et al., 2022; Jimenez et al., 2020).

Religious beliefs can facilitate or inhibit the adoption of better health-related attitudes in the context of health behavior. If individuals believe that an illness is a test from God, they will believe that God is on their side and that they can accept the illness. (Sukkarieh-Haraty et al., 2019). Another approach to fatalism is to believe that illnesses are given by God, and that whatever a person does will not change what is going to happen regarding that illness. This is why medicine, religion, and fatalism are intertwined. Consequently, fatalism may have positive or negative effects on health-seeking behavior in combination with religious beliefs. (Morgan et al., 2008; Ramírez, 2014).

The twin earthquakes that occurred in Türkiye on February 6, 2023, proved to be one of the most painful experiences of humankind in this century. The losses caused by the earthquakes, the destruction of living spaces, and the creation of a chaotic atmosphere in the surrounding area had a profound effect on the people who experienced these two earthquakes. In the study carried out by Kirman (2023) on the experiences of the victims of the earthquakes of February 2023 in Türkiye, it was found that the individuals most often linked the circumstances to religion and referred to them as the will of God, in other words as fate. (Kirman, 2023). On the other hand, the negative conditions that arose in the period following the earthquake also had a negative impact on the health of individuals. Ahmed et al. (2023) found that in the period following the earthquake, individuals experienced a psychological breakdown, suffered from malnutrition, and were exposed to the risks of chronic diseases. Thus, in the Turkish society where religious beliefs are dominant, all of these factors, combined with a fatalistic approach, may have an impact on the healthy lifestyle behaviors of individuals.

The relevant literature includes studies on earthquakes and fatalistic perceptions (Abbott, 2020; Sun et al., 2022), and studies on the impact of earthquakes on social health. (Ahmed et al., 2023; Cohn, 2015; Hussain et al., 2011; Davis et al., 2010). However, to the best of our knowledge, there is no study that has investigated the relationship between the fatalistic approaches and the healthy lifestyle behavior of the earthquake victims. Following the earthquakes in Türkiye, a large number of people were affected socially, physically, environmentally, and psychologically in the short and long term. It was observed that in order to make sense of what they had experienced, earthquake victims first made statements with religious connotations and sought refuge in faith. (Kafalı, 2023; Kirman, 2023).

In this process, it is important to address the relationship between fatalistic attitudes and health behaviors of earthquake victims. Therefore, the aim of this study is to analyze the relationship between health fatalism and healthy lifestyle behaviors among earthquake victims following the recent earthquakes in Türkiye.

## Research Questions

1. What are the religious health fatalism levels in earthquake victims?

2. What are the healthy lifestyle behaviors of earthquake victims?

3. Is there a statistically significant relationship between religious health fatalism and healthy lifestyle behaviors in earthquake victims?

4. Is there a statistically significant difference between the mean scores of healthy lifestyle behaviors according to the sociodemographic characteristics of earthquake victims?

## Methods

### Type of Study

This study is descriptive, cross-sectional, and correlational.

### Sample

In this study, G*Power 3.1.9.4 was utilized to calculate the minimum sample size required, while its “Correlation: Bivariate normal model” was selected for the correlation analysis. The minimum sample size was calculated with a statistical power of 95%, a type-1 error margin of 5%, and a correlation coefficient of 0.3 recommended by the software. According to these calculations, the minimum sample size was found to be 138.

The study covers individuals affected by earthquakes in southeast Türkiye in 2023. In the post-earthquake period, many earthquake victims migrated to safe regions across Türkiye and were placed in student dormitories, while their needs (including accommodation, nutrition, clothing, health screenings, education, physical exercise, psychosocial support) were met by the Turkish government.

The population of this study included earthquake victims temporarily staying in a student dormitory in a province in northern Türkiye. Individuals were invited to participate by personally filling out a structured validated questionnaire. A total of 358 earthquake victims meeting the definition of the population were staying in the dormitory. The sample of the study consists of 175 earthquake survivors who voluntarily accepted to participate in the research. The rate of participation in the study was 48.8%. Additionally, this study followed Strengthening the Reporting of Observational Studies in Epidemiology (STROBE; Vandenbroucke et al., 2007) guidelines for cross-sectional studies.

### Place and Time of Study

The study was carried out at the dormitory where the earthquake victims were lodging from June 15 to July 6, 2023.

### Inclusion and Exclusion Criteria

Volunteering to take part in the study, being at least 18 years old, and not having any cognitive or communicative impairments were the inclusion criteria. Only people affected by the major earthquake in southeast Türkiye in 2023 were included in the study. The research did not include earthquake victims who immigrated to Türkiye from other countries and were unable to converse in Turkish.

### Dependent and Independent Variables

Healthy lifestyle behaviors and religious health fatalism are the study’s dependent variables. The sociodemographic and earthquake-related traits of earthquake victims are the independent variables.

### Measures

The data were collected using an Individual Characteristics Form, the Religious Health Fatalism Questionnaire, and the Healthy Lifestyle Behaviors Scale-II.

#### Individual Characteristics Form

This form was created by the researchers, and its first part contained nine questions about each participant’s age, gender, socioeconomic status, occupation, and chronic disease status, while its second part consisted of four questions about each participant’s pre-earthquake health state, being injured in the earthquakes, being trapped under earthquake debris, and having house damaged due to the earthquakes.

#### Religious Health Fatalism Questionnaire (RHFQ)

The RHFQ was developed by Franklin et al. (2007) to identify whether religious health fatalism was associated, in a general sense, with health behaviors. In 2018, Bobov and Capik (2020) conducted a validity and reliability study for the Turkish form of the RHFQ. Designed as a five-point Likert-type scale, the RHFQ comprises 17 items, and the total RFHQ score ranges from 17 points to 85. Higher RHFQ scores are associated with increased fatalistic tendencies (Table 1). Cronbach’s alpha coefficient was found as 0.91 for the original version of the RHFQ (Bobov & Capik, 2020). In this study, Cronbach’s alpha coefficient was calculated as 0.93.

**Table 1:** Religious Health Fatalism Questionnaire (Bobov & Capik, 2020)

	Below are the items related to health fatalism. Please tick the statement that suits you between Strongly Disagree and Strongly Agree. Please tick only one option for each item	(1) Strongly Disagree	(2) Disagree	(3) Undecided	(4) Agree	(5) Strongly Agree
1	If I just pray to God about my health, He will work it out	1	2	3	4	5
2	When I am sick I give my burdens to God and let Him handle it	1	2	3	4	5
3	God will take care of my health because I have found favor in his sight	1	2	3	4	5
4	If God wants me to have better health, He will provide	1	2	3	4	5
5	I don’t worry about my health because it is in God’s hands	1	2	3	4	5
6	If I am sick, I have to wait until it is God’s time for me to be healed	1	2	3	4	5
7	When I have a health problem, I pray for God’s will to be done	1	2	3	4	5
8	As long as I stay focused in prayer, I will be healed of any sickness	1	2	3	4	5
9	Spiritual people should accept whatever God has meant for them	1	2	3	4	5
10	I trust God, not man, to heal me	1	2	3	4	5
11	If a person has enough faith, healing will occur without doctors having to do anything	1	2	3	4	5
12	Sometimes, God allows people to be sick for a reason	1	2	3	4	5
13	If I become ill, God intended that to happen	1	2	3	4	5
14	Whatever illnesses I will have, God has already planned it	1	2	3	4	5
15	Sometimes someone can be ill because of disobedience to God	1	2	3	4	5
16	I don’t need to try to improve my health because I know it is up to God	1	2	3	4	5
17	I can control a small health issue, but only God can control a big health issue	1	2	3	4	5

Place Table 1 about here.

#### Healthy Lifestyle Behaviors Scale-II (HLBS-II)

The HLBS is a Likert-type scale developed by Walker et al. in 1987. It was revised in 1996 as the Healthy Lifestyle Behaviors Scale-II (HLBS-II). Bahar et al. (2008) performed the validity and reliability study for the Turkish form of the HLBS-II. The HLBS-II contains 52 items, and each of its items is rated from 1 to 4. All HLBS-II items are positively worded. The subscales of the HLBS-II are health responsibility (nine items), physical activity (eight items), nutrition (nine items), spiritual development (nine items), interpersonal relationships (nine items), and Stress Management (eight items). The minimum and maximum scores to be obtained from the HLBS-II are 52 and 208. It is assumed that, as the total HLBS-II score increases, the individual exhibits more healthy lifestyle behaviors. In the study by Bahar et al. (2008), Cronbach’s alpha coefficient was reported as 0.92 for the HLBS-II. This coefficient was the same in our study (Bahar et al., 2008).

### Data Collection

After receiving the permissions required to conduct the study, the data collection stage was initiated. In the dormitory where earthquake victims temporarily stayed, the researchers informed earthquake victims about the topic and method of the study, and the participation being voluntary. Those who agreed to participate in the study were included. It took 7–10 min for each participant to fill out the data collection forms.

### Ethical Approval

To conduct the study, the researchers obtained ethical approval from the Non-Invasive Clinical Trials Ethics Committee of a public university in Türkiye (Date: June 9, 2023, No: E-26428519–044-86479). Earthquake victims were informed about the study and ensured that participation in the study was voluntary, and the confidentiality of their data to be shared with the researchers would be protected. They were also informed that they were free to withdraw from the study anytime they like, and in return, they verbally expressed their consent to participate in the study. This study was carried out in full compliance with the ethical standards set out in the Declaration of Helsinki. All earthquake victims volunteering to participate in the study were included in the sample, and the confidentiality of their data was fully respected.

### Statistical Analysis of Data

Data were analyzed using the Statistical Package for the Social Sciences (SPSS) 25.0 software package. The data were found to be acceptable for a normal distribution after the Kolmogorov–Smirnov test was performed to evaluate the data to see whether they were normally distributed. Percentages, mean values, and standard deviations were used as descriptive statistics. As the data were normally distributed, an independent sample t test was used to compare two groups, while one-way analysis of variance (ANOVA) was utilized to compare more than two groups. Post hoc analyses were conducted to identify the sources of difference when a significant difference was determined in the multi group comparison. The relationship between the scale scores of the participants was analyzed via Pearson’s correlation test as the data were normally distributed. In the correlation analysis, the correlation coefficient (r), which ranges from -1 to 1, was interpreted based on its value (r = 0.00: no relationship, r = 0.01–0.29: a weak relationship, r = 0.30–0.70: a moderate relationship, r = 0.71–0.99: a strong relationship, r = 1.00: a very strong relationship) (Schober et al., 2018). In addition, multiple regression analysis was used to assess the effect of the independent variables on the total RHFQ and HLBS-II scores. In this study, the statistical significance was identified if the p value was below 0.05 (p < 0.05). There are no missing data in the study.

## Results

All participants who met the inclusion criteria of the study completed the forms in full. The data obtained from 175 earthquake victims were analyzed (n = 175).

### Sociodemographic Characteristics

The mean age of the participants was 36.06 years (standard deviation [SD] = 16.34) with particular ages ranging from 17 to 79 years. It was determined that 56.6% of the participants were female, 42.9% held university degrees, 57.1% were married, 46.3% had low levels of income, and 50.9% had no children. A large majority of the participants had no chronic diseases (65.7%). Most participants stated that they were not smokers (62.3%) and did not consume alcohol (92.6%) (Table 2).

**Table 2:** Sociodemographic Characteristics (n = 175)

Characteristics	Categories	*n* (%)	Mean (*SD*)	Range
Age (years)			36.06 (16.34)	17–79
Gender	Female	99 (56.6)		
	Male	76 (43.4)		
Education status	Illiterate	16 (9.1)		
	Elementary school	23 (13.1)		
	Middle school	20 (11.4)		
	High school	41 (23.5)		
	University	75 (42.9)		
Marital status	Married	100 (57.1)		
	Single	75 (42.9)		
Income status	Income below expenses	81 (46.3)		
	Income equal to expenses	80 (45.7)		
	Income above expenses	14 (8.0)		
Has children	Yes	86 (49.1)		
	No	89 (50.9)		
Chronic disease	None	115 (65.7)		
	Hypertension	16 (9.1)		
	Diabetes	14 (8.0)		
	Asthma	14 (8.0)		
	Others (chronic renal failure, cancer, and heart failure)	16 (9.1)		
Smoker	Yes	66 (37.7)		
	No	109 (62.3)		
Consumes alcohol	Yes	13 (7.4)		
	No	162 (92.6)		

Place Table 2 about here.

### Earthquake-Related Characteristics

Table 3 shows the earthquake-related characteristics of the participants. 34.9% of the participants stated that their general health state was almost the same as before the earthquakes. Some of the participants experienced minor damage to their houses due to the earthquakes (33.1%) or were injured in the earthquakes (14.9%) (Table 3).

**Table 3:** Earthquake-related Characteristics (n = 175)

Characteristics	Categories	n (%)
Perceived general health status in comparison with the pre-earthquake period	Better	19 (10.9)
	Slightly better	50 (28.6)
	Almost the same	61 (34.9)
	Slightly worse	45 (25.6)
Status of having damage in the house due to earthquakes	No damage	31 (17.7)
	Little damage	58 (33.1)
	Moderate damage	39 (22.3)
	Heavy damage	37 (21.1)
	Destroyed	10 (5.8)
Was trapped under earthquake debris	Yes	17 (9.7)
	No	158 (90.3)
Was injured in the earthquakes	Yes	26 (14.9)
	No	149 (85.1)

Place Table 3 about here.

### Earthquake Victims' Scale Scores

As seen in Table 4, the mean RHFQ score of the participants was 54.89 ± 14.68. The mean scores of the participants on the overall HLBS-II and its health responsibility, physical activity, nutrition, spiritual development, interpersonal relationships, and Stress Management subscales were 130.14 ± 19.68 and 22.78 ± 4.3, 16.73 ± 3.93, 19.89 ± 3.84, 26.0 ± 4.83, 25.3 ± 4.49, and 19.41 ± 3.68, respectively (Table 4).

**Table 4:** Mean, Minimum, and Maximum RHFQ and HLBS-II Scores

Scales and Subscales		Minimum	Maximum	Mean	Standard deviation
RHFQ		17	85	54.89	14.68
HLBS-II		78	186	130.14	19.68
1	Health responsibility	12	34	22.78	4.3
2	Physical activity	9	30	16.73	3.93
3	Nutrition	10	30	19.89	3.84
4	Spiritual development	12	36	26.0	4.83
5	Interpersonal relationships	11	36	25.3	4.49
6	Stress Management	11	29	19.41	3.68

Place Table 4 about here.

### Comparison of Earthquake Victims’ Sociodemographic Characteristics and Scale Scores

Table 5 presents the results of the comparisons of the mean RHFQ and HLBS-II scores of the participants based on their sociodemographic variables. We determined that their mean RHFQ and HLBS-II scores varied significantly based on their age, marital status, and status of having children (p < 0.05). Besides, there were statistically significant differences in the mean RHFQ scores of the participants based on their education levels, income status, chronic disease occurrence, alcohol consumption status, perceived general health status in comparison with the pre-earthquake period, experiencing damage to their house due to the earthquakes, and being trapped under earthquake debris (p < 0.05) (Table 5).

**Table 5:** Comparison of Mean RHFQ and HLBS-II Scores Based on Sociodemographic Characteristics

Variables	Categories	RHFQ	HLBS-II
Age		0.534^a^ p: 0.001*	0.249^a^ p: 0.001*
		*M* (*SD*)	*M* (*SD*)
Gender	Female	54.39 (14.78)	130.32 (20.22)
	Male	55.55 (14.63)	129.90 (19.10)
		-0.516^b^ p: 0.6	0.138^b^ p: 0.8
Education status	Illiterate ^1^	68.68 (12.93)	136.25 (13.84)
	Elementary school ^2^	62.73 (8.50)	137.73 (19.41)
	Middle school ^3^	60.05 (9.76)	128.30 (14.44)
	High school ^4^	55.26 (12.41)	125.12 (23.51)
	University ^5^	47.97 (15.09)	129.74 (19.15)
		12.61^c^ *p* = 0.001* 5 < 1,2,3	2.004^c^ *p* = 0.96
Marital status	Married	60.93 (10.86)	134.36 (18.40)
	Single	46.85 (15.31)	124.52 (20.05)
		6.782^b^ *p* = 0.001*	3.368^b^ *p* = 0.001*
Income status	Income below expenses ^1^	58.35 (14.01)	126.56 (18.86)
	Income equal to expenses ^2^	51.0 (14.31)	133.03 (19.99)
	Income above expenses ^3^	57.14 (16.08)	134.28 (20.49)
		5.499^c^ *p* = 0.005* 1 > 2	2.555^c^ *p* = 0.08
Has children	Yes	61.53 (10.50)	135.59 (17.30)
	No	48.48 (15.33)	124.87 (20.49)
	6.587^b^ *p* = 0.001*	3.731^b^ *p* = 0.001*	
Chronic disease	None ^1^	52.70 (14.13)	130.11 (19.45)
	Hypertension ^2^	60.37 (12.33)	135.75 (15.29)
	Diabetes ^3^	61.42 (12.52)	127.64 (23.51)
	Asthma ^4^	60.42 (15.14)	122.85 (19.28)
	Others ^5^ (Chronic renal failure, cancer, heart failure)	54.62 (18.98)	133.31 (22.02)
		2.468^c^ *p* = 0.047^*^ 1 < 2,3,4	0.963^c^ *p* = 0.42
Smoker	Yes	56.30 (14.19)	132.77 (19.83)
	No	54.04 (14.97)	128.55 (19.51)
		0.985^b^ *p* = 0.32	1.37^b^ *p* = 0.17
Consumes alcohol	Yes	46.92 (18.99)	128.61 (24.79)
	No	55.53 (14.16)	130.26 (19.31)
		-2.053^b^ *p* = 0.04*	-0.29^b^ *p* = 0.77
Perceived general health status in comparison with the pre-earthquake period	Better ^1^	58.47 (12.13)	130.57 (14.96)
	Slightly better ^2^	56.76 (14.45)	129.74 (20.76)
	Almost the same ^3^	49.52 (15.13)	132.01 (20.12)
	Slightly worse ^4^	58.60 (13.57)	127.86 (19.96)
		4.58^c^ *p* = 0.04* 4 > 3	0.391^c^ *p* = 0.76
Status of having damage in the house due to the earthquakes	No damage ^1^	48.77 (16.44)	131.32 (17.28)
	A little damaged ^2^	52.15 (14.44)	132.01 (20.94)
	Moderately damaged ^3^	59.05 (12.95)	126.12 (21.55)
	Heavily damaged ^4^	61.13 (12.46)	133.27 (16.47)
	Demolished ^5^	50.50 (13.53)	119.70 (20.34)
		4.934^c^ *p* = 0.001* 1 < 3,4	1.519^c^ *p* = 0.19
Was trapped under earthquake debris	Yes	61.11 (12.70)	128.35 (23.2)
	No	53. 90 (14.57)	130.33 (19.34)
		2.776^b^ *p* = 0.006*	-0.394^b^ *p* = 0.69
Was injured in the earthquakes	Yes	56.46 (14.37)	126.23
	No	54.10 (14.64)	130.82 (19.82)
		1.727^b^ *p* = 0.08	-1.099^b^ *p* = 0.27

^*^p < 0.05, ^a^Pearson’s correlation coefficient, ^b^Independent-samples t test, ^c^One-way ANOVA test *M*: Mean, *SD*: Standard deviation

Place Table 5 about here.

### Correlation of Scale Scores

Table 6 presents the results of the correlation analysis between the RHFQ and HLBS-II scores of the participants. There was a weak, positive, and statistically significant relationship between the RHFQ and HLBS-II scores of the participants (r = 0.223) (p < 0.05). Weak, positive, and statistically significant relationships were also identified between the RHFQ scores of the participants and their HLBS-II health responsibility (r = 0.216), nutrition (r = 0.252), spiritual development (r = 0.210), interpersonal relationships (r = 0.178), and Stress Management (r = 0.243) subscale scores (p < 0.05) (Table 6).

**Table 6:** Correlations Between RHFQ and HLBS-II Scores

Variables			RHFQ
HLBS-II		r	0.223
		*p*	0.003*
HLBS-II Subscales	Health responsibility	*r*	0.216
		*p*	0.004*
	Physical activity	*r*	-0.059
		*p*	0.43
	Nutrition	*r*	0.252
		*p*	0.001*
	Spiritual development	*r*	0.210
		*p*	0.005*
	Interpersonal relationships	*r*	0.178
		*p*	0.018*
	Stress Management	*r*	0.243
		*p*	0.001*

*r* = Pearson’s correlation coefficient, *p < 0.05

Place Table 6 about here.

### Multiple Regression Analysis

Table 7 shows the results of multiple regression analyses of sociodemographic characteristics affecting the RHFQ and HLBS-II scores. It was determined that the independent variables explained 35.7% of the RHFQ scores (R2 = 0.357). Accordingly, it was found that the age, education status, and chronic disease of the participants had a statistically significant effect on the total scale score (p < 0.05). Sociodemographic variables explained 16% of the HLBS-II scale score (R2 = 0.160). Among these variables, age and income status had a significant effect on the scale score (p < 0.05).

**Table 7:** Multiple Regression Analysis of Sociodemographic Characteristics Affecting RHFQ and HLBS-II Scores

Ölçek	Variables	B	SE	β	t	p
RHFQ	Age	0.307	0.097	0.342	3.172	0.002*
	Gender	-0.670	2.170	-0.023	-0.309	0.758
	Education status	-2.530	0.992	-0.234	-2.550	0.012*
	Marital status	-4.979	3.221	-0.168	-1.546	0.124
	Income status	-0.893	1.520	0.038	-0.588	0.558
	Has children	0.292	3.239	0.010	0.090	0.928
	Chronic disease	4.867	2.410	0.156	2.020	0.045*
	Smoker	-1.981	2.100	-0.066	-0.943	0.347
	Consumes alcohol	3.779	3.653	0.068	1.034	0.303
	R = 0.597, R2 = 0.357, F = 10.166, p = 0.001					
HLBS-II	Age	0.340	0.148	0.282	2.292	0.023*
	Gender	-5.556	3.324	-0.140	-1.671	0.097
	Education status	0.903	1.520	0.062	0.594	0.553
	Marital status	1.892	4.933	0.048	0.383	0.702
	Income status	6.933	2.328	0.222	2.979	0.003*
	Has children	-9.452	4.960	-0.241	-1.905	0.058
	Chronic disease	4.736	3.691	0.113	1.283	0.201
	Smoker	-5.357	3.217	-0.132	-1.665	0.098
	Consumes alcohol	-0.994	5.596	-0.013	-0.178	0.859
	R = 0.400, R^2^ = 0.160, F = 3.49, p = 0.001					

Statistical significance in this analysis is shown in bold

^*^p < 0.05

Place Table 7 about here.

## Discussion

Fatalism is a basic phenomenon that is encountered in almost all spheres of social life and can influence human life. This tendency can be prevalently observed in several geographic locations with inadequate socioeconomic development. This situation can be observed in Türkiye as well. Particularly, fatalism is in a dynamic position that is likely to influence people’s intentions to act in the aftermath of incidents occurring in their lives. Studies indicated that following natural disasters, the fatalistic tendencies of people surface based on their religious beliefs (Kartopu, 2013; Sun et al., 2022). Besides, fatalism was examined as a barrier to participation in health improvement programs and the utilization of health services (Franklin et al., 2007). This study analyzed the relationship between religious health fatalism tendencies and healthy lifestyle behaviors in victims of the two large-scale earthquakes occurring consecutively in Türkiye in February 2023.

According to the results of this study, the mean RHFQ score of the participants was 54.89 ± 14.68. Considering the minimum and maximum possible scores to be obtained from the RHFQ (17–85), it can be asserted that the participants achieved above-average RHFQ scores in general. In the relevant literature, there is no study examining the religious health fatalism levels of earthquake victims directly. However, there are studies analyzing fatalistic tendencies in different groups. In the study performed by Solmaz and Durmaz (2023) with the participation of individuals who previously had COVID-19 and individuals who were never infected with COVID-19, it was found that the mean RHFQ score of the participants was above-average (Solmaz & Durmaz, 2023). Moreover, in studies conducted with patients diagnosed with hypertension, results similar to our findings were obtained (Gutierrez et al., 2017; Özer & Turan, 2023). In Turkish society, those who believe that circumstances such as diseases, epidemics, and natural disasters are given by God form a large majority. This may result from the fact that nearly all Turkish people are Muslims. In a study in Türkiye, it was found that almost half of the participants believed that there was little they could do to change their lives (Dayapoglu, 2021). There have been studies of religious health fatalism in different countries. The results of a few studies show that participants in the sample group said that religion has an effect on health. The results of the studies showed that religious fatalism may be effective in the management of chronic diseases and refusal of vaccines (Alyazidi et al., 2022; de Dios et al., 2020; Mamani-Benito et al., 2023; Wong et al., 2022). In the study conducted by Alyazidi et al. with health sciences students, it was determined that the highest fatalism scores were found in Muslim and Christian students. The result of our study resembled those of previous studies. This result is an indication of society's view of religion as an important factor in the management of disease conditions. One reason for this may be the fact that all of them were conducted in Türkiye.

Next, in this study, it was found that the HLBS-II scores of the participants were moderate in general. Additionally, their HLBS-II physical activity and nutrition subscale scores were below-average in general, and they had above-average scores on the HLBS-II spiritual development and interpersonal relationships subscales. The 2023 earthquakes described as one of the greatest disasters of the century in Türkiye prevented the satisfaction of certain physiological and psychological needs known as basic human needs, such as housing, food, and security. This situation led to problems in practices related to the health of earthquake victims. Kuşçu and Göde (2023) found that associate degree students receiving health education had moderately healthy lifestyle behaviors in the aftermath of the large-scale Türkiye earthquakes (Kuşçu & Göde, 2023). In the study by Bell et al. (2019), older people were identified to exhibit negative health behaviors such as an increase in weight gain and a decrease in physical activity (Bell et.al., 2019). Thus, studies indicated that earthquakes could affect the health-related behaviors of individuals. This situation may have resulted from the fact that places, where individuals used to perform physical activities, were demolished because of the damage inflicted by the earthquakes. The higher HLBS-II spiritual development and interpersonal relationships subscale scores of the participants relative to their other HLBS-II subscale scores can be explained by the possibility that they adopted religious belief systems and the social environment to a higher extent as a coping strategy to respond to unforeseen life incidents.

Moreover, in this study, it was discerned that the religious health fatalism scores of the participants increased as their age increased. It was also found that the participants who had low education levels, those who were married, those who had low levels of income, and those who had children had higher mean religious health fatalism scores than other respective groups. In addition, age, educational status, and chronic disease of the participants affected the total scale score of religious health fatalism. In the study that Bobov and Capik (2020) conducted to test the validity and reliability of the RHFQ in Turkish, it was stated that fatalistic tendencies were higher at advanced ages. On the other hand, the results of certain studies indicated that there was no statistically significant relationship between age and fatalistic tendencies (Keller et al., 2021; Özer & Turan, 2023). The result of this study may have been affected by the possibility that there is an increase in religious beliefs along with aging, and in this process, fatalism becomes more dominant with the increase in age. In studies conducted in Türkiye on health fatalism, it was put forward that fatalism scores increased as education levels decreased (Bobov & Capik, 2020; Özer et al., 2022; Solmaz & Durmaz, 2023). The result of our study was in a similar vein to the results of other studies. The increase in education levels adds a different perspective to individuals and helps them solve their problems effectively. Thus, it is considered that the participants of this study who had low levels of education may have exhibited a fatalistic attitude by turning to religion for their health and did not search for different solutions. Besides, in studies conducted in Türkiye on individuals diagnosed with cancer, epilepsy, and breast cancer, it was found that married individuals had higher fatalism scores (Solmaz & Durmaz, 2023). The result of this study was similar to those in the relevant literature. Marriage is a situation that increases an individual’s responsibilities and requires them to hold on to life more firmly. It is thought that this situation could produce stress for married individuals considering the responsibilities of married individuals toward their spouses and their children and in this sense, married individuals may exhibit a fatalistic approach toward coping with this stress.

Furthermore, the results of this study show that the participants who perceived their general health state as slightly worse in comparison with the pre-earthquake period had relatively high religious health fatalism scores. The participants whose houses were heavily damaged by the earthquakes and those who had been trapped under earthquake debris also had relatively high religious health fatalism scores. This result can be explained by the relationship between fatalism and defense mechanisms. Individuals may tend to avoid responsibility to protect themselves in the struggle to cope with incidents that they cannot overcome. Hence, they may embrace a firmly fatalistic approach. Individuals take refuge in fatalism to evade their responsibilities and resort to self-deception by attributing situational responsibilities to a supernatural power (Kaya & Bozkur, 2017).

Besides, in this study, it was discerned that the participants exhibited healthier lifestyle behaviors as their age increased. The absence of studies analyzing the healthy lifestyle behaviors of victims of earthquakes or other disasters in the relevant literature makes it difficult to discuss the results of this study. In our study, age had an effect on the total score of healthy lifestyle behaviors in our study. However, in some studies conducted during the COVID-19 pandemic, it was found that age affected healthy lifestyle behaviors, and scores for behaviors such as health responsibility, physical activity, and nutrition increased, particularly in advanced age. The results of these studies were similar to those obtained in our study, and this situation shows that more importance is attributed to health along with an increase in age.

Additionally, in this study, the participants who were married and those who had children enjoyed significantly higher mean healthy lifestyle behavior scores comparing to other respective groups. In our study, marital status had an effect on the total score of healthy lifestyle behaviors in our study. Even if it is harder for individuals to continue healthy lifestyle behaviors such as having a healthy diet, sleeping enough, coping with stress, and doing physical exercise after disasters like earthquakes, the increased responsibility of earthquake victims who are married and have children necessitates the continuation of these behaviors. The result obtained in our study may also be explained by the possibility that having children imposes more responsibilities on families, and families pay more attention to healthy lifestyle behaviors, especially for the healthy development of their children.

Lastly, in this study, we have spotted a weak, positive, and statistically significant relationship between the religious health fatalism levels and healthy lifestyle behaviors of the participants. It was further discerned that religious health fatalism had weak, positive, and statistically significant relationships to health responsibility, nutrition, spiritual development, interpersonal relationships, and stress management. In the study conducted by Kuh and Erdem (2019) to examine fatalistic tendencies and healthy lifestyle behaviors, positive relationships were identified between scores of certain subscales of the Fatalism Tendency Scale and scores of HLBS-II nutrition, spiritual development, and interpersonal relationships subscales (Kuh & Erdem, 2019). In the study performed by Martin et al. (2015) to analyze the relationship between fatalism and healthy lifestyle behaviors in individuals diagnosed with cardiovascular diseases and living in rural areas, it was found that attachment to healthy lifestyle behaviors increased as fatalism increased (Martin et al., 2015). As individuals believing in fatalism assume that their bodies are entrusted to them by God, they may try to stay away from conditions that threaten their health. This situation may have encouraged individuals to adopt healthy lifestyle behaviors.

### Limitations

The study we conducted has some limitations. The data for this study were gathered from earthquake survivors who temporarily relocated to the north following two severe earthquakes in eastern Türkiye. Since the study’s findings are based on the accounts of a small sample of earthquake victims, they cannot be applied to all earthquake victims. In addition, it is thought that the spiritual values and culture of earthquake victims may be effective on religious health fatalism.

## Conclusion

It was concluded that earthquake victims had above-average religious health fatalism tendencies. The participants also exhibited moderately healthy lifestyle behaviors. There was a weak positive relationship between the religious health fatalism tendencies and healthy lifestyle behaviors of the participants. More research is needed to identify how the fatalistic tendencies of individuals from different cultures affect the health behaviors and health outcomes of these individuals in the context of natural disasters such as earthquakes, floods, landslides, and whirlwinds. Qualitative and quantitative studies on the health behaviors and fatalistic tendencies of individuals should be conducted not only after natural disasters but also before natural disasters occur. If fatalism is linked to religious beliefs, it can be more firmly adopted by individuals. There is a need to research the factors affecting the health practices of society in the context of religious beliefs and fatalism. In the future, it should be done with individuals affected by earthquakes in different regions of Türkiye and the sample should be expanded.
